# The SK4 channel allosteric blocker, BA6b9, reduces atrial fibrillation substrate in rats with reduced ejection fraction

**DOI:** 10.1093/pnasnexus/pgae192

**Published:** 2024-05-12

**Authors:** Shira Burg, Or Levi, Sigal Elyagon, Shir Shapiro, Michael Murninkas, Sharon Etzion, Gideon Gradwohl, Daria Makarovsky, Alexandra Lichtenstein, Yaara Gordon, Bernard Attali, Yoram Etzion

**Affiliations:** Department of Physiology & Pharmacology, Sackler Faculty of Medicine and Sagol School of Neurosciences, Tel Aviv University, Tel Aviv 69978, Israel; Cardiac Arrhythmia Research Laboratory, Department of Physiology and Cell Biology, Faculty of Health Sciences, Ben-Gurion University of the Negev, Beer-Sheva 8410501, Israel; Regenerative Medicine & Stem Cell Research Center, Ben-Gurion University of the Negev, Beer-Sheva 8410501, Israel; Cardiac Arrhythmia Research Laboratory, Department of Physiology and Cell Biology, Faculty of Health Sciences, Ben-Gurion University of the Negev, Beer-Sheva 8410501, Israel; Regenerative Medicine & Stem Cell Research Center, Ben-Gurion University of the Negev, Beer-Sheva 8410501, Israel; Cardiac Arrhythmia Research Laboratory, Department of Physiology and Cell Biology, Faculty of Health Sciences, Ben-Gurion University of the Negev, Beer-Sheva 8410501, Israel; Regenerative Medicine & Stem Cell Research Center, Ben-Gurion University of the Negev, Beer-Sheva 8410501, Israel; Cardiac Arrhythmia Research Laboratory, Department of Physiology and Cell Biology, Faculty of Health Sciences, Ben-Gurion University of the Negev, Beer-Sheva 8410501, Israel; Regenerative Medicine & Stem Cell Research Center, Ben-Gurion University of the Negev, Beer-Sheva 8410501, Israel; Regenerative Medicine & Stem Cell Research Center, Ben-Gurion University of the Negev, Beer-Sheva 8410501, Israel; Medical Engineering Unit, The Jerusalem College of Technology, Jerusalem 9116001, Israel; Inter-Departmental Core Facility, Sackler Faculty of Medicine, Tel Aviv University, Tel Aviv 69978, Israel; Inter-Departmental Core Facility, Sackler Faculty of Medicine, Tel Aviv University, Tel Aviv 69978, Israel; Department of Clinical Microbiology and Immunology, Sackler Faculty of Medicine, Tel Aviv University, Tel Aviv 69978, Israel; Department of Physiology & Pharmacology, Sackler Faculty of Medicine and Sagol School of Neurosciences, Tel Aviv University, Tel Aviv 69978, Israel; Cardiac Arrhythmia Research Laboratory, Department of Physiology and Cell Biology, Faculty of Health Sciences, Ben-Gurion University of the Negev, Beer-Sheva 8410501, Israel; Regenerative Medicine & Stem Cell Research Center, Ben-Gurion University of the Negev, Beer-Sheva 8410501, Israel

**Keywords:** SK4 channel, KCa3.1, atrial fibrillation, atrial remodeling, HFrEF

## Abstract

Atrial fibrillation (AF), the most common cardiac arrhythmia, is strongly associated with several comorbidities including heart failure (HF). AF in general, and specifically in the context of HF, is progressive in nature and associated with poor clinical outcomes. Current therapies for AF are limited in number and efficacy and do not target the underlying causes of atrial remodeling such as inflammation or fibrosis. We previously identified the calcium-activated SK4 K^+^ channels, which are preferentially expressed in the atria relative to the ventricles in both rat and human hearts, as attractive druggable target for AF treatment. Here, we examined the ability of BA6b9, a novel allosteric inhibitor of SK4 channels that targets the specific calmodulin-PIP2 binding domain, to alter AF susceptibility and atrial remodeling in a systolic HF rat postmyocardial infarction (post-MI) model. Daily BA6b9 injection (20 mg/kg/day) for 3 weeks starting 1-week post-MI prolonged the atrial effective refractory period, reduced AF induction and duration, and dramatically prevented atrial structural remodeling. In the post-MI left atrium (LA), pronounced upregulation of the SK4 K^+^ channel was observed, with corresponding increases in collagen deposition, α-SMA levels, and NLRP3 inflammasome expression. Strikingly, BA6b9 treatment reversed these changes while also significantly reducing the lateralization of the atrial connexin Cx43 in the LA of post-MI rats. Our findings indicate that the blockade of SK4 K^+^ channels using BA6b9 not only favors rhythm control but also remarkably reduces atrial structural remodeling, a property that is highly desirable for novel AF therapies, particularly in patients with comorbid HF.

**Significance Statement**

Atrial fibrillation (AF) and heart failure (HF) are two challenges of major significance. AF has a progressive nature especially in HF patients. Current treatment modalities are limited and do not target the development of inflammation and fibrosis in the tissue. Recent data indicate that SK4 calcium-activated K^+^ channels are expressed predominantly in supraventricular cardiomyocytes. In addition, these channels are overexpressed in activated fibroblasts and macrophages. Here, we found that BA6b9, a novel allosteric blocker inhibiting SK4 channels, markedly inhibits AF susceptibility and atrial inflammation and fibrosis in rats with systolic HF following myocardial infarction. Thus, blockade of SK4 K^+^ channels by BA6b9 may represent an attractive new strategy to inhibit AF development and progression in patients suffering from systolic HF.

## Introduction

Atrial fibrillation (AF) is the most common cardiac arrhythmia, affecting 1–2% of the population worldwide (1, 2). The prevalence of AF steeply increases with age and the lifetime risk is estimated to be as high as 22–26% (3). AF is characterized by rapid and disordered electrical activation of the atria leading to compromised contraction. It is associated with higher rates of stroke, heart failure (HF), hospital admissions, and mortality, thus representing an important medical challenge (4). Common conditions such as ischemic heart disease and HF with either reduced ejection fraction (EF) or preserved EF (HFrEF and HFpEF, respectively) are important etiological factors for AF (5, 6), and they converge together with additional etiological factors such as hypertension, obesity, diabetes mellitus, and sleep apnea to increase the risk of AF in ways that remain elusive (7). AF is a progressive condition that results in a greater risk of recurrence and persistence over time, complicating treatment efforts (8). Importantly, when associated with HF, AF leads to poor clinical outcomes, and the available pharmacological treatment options for rhythm control are limited (9, 10).

The pathophysiology of AF is complex and multifactorial. AF initiation involves sources of sustained rapid electrical activity (“drivers”) that can trigger arrhythmic episodes (11). In addition, various mechanisms may alter the electrical and structural properties of the atria and thus promote tissue susceptibility to AF episodes (“AF substrate”) (1, 8). AF itself can also induce atrial electrical and structural remodeling, thereby increasing the AF substrate (8, 12). Antiarrhythmic drugs aimed at preventing AF recurrence and sustaining normal sinus rhythm are extensively used but are only partially effective and are not devoid of proarrhythmic or extra-cardiac side effects (8, 13–15). Novel atrial selective antiarrhythmic targets have been the focus of extensive research over the last two decades, albeit without success in translating these efforts to advanced clinical studies to date (15, 16). From a different perspective, therapeutic modalities intended to attenuate or revert underlying pathophysiological processes that promote AF substrate are attractive potential adjuncts to current management strategies. Various insults such as inflammation, oxidative and endoplasmic reticulum stress, abnormal proteostasis, apoptotic signaling, autophagy, and fibrosis are all considered possible drug targets in this context (17–21).

We previously identified a new druggable target in the heart in the form of the calcium-activated SK4 K^+^ channels, the existence of which had previously been overlooked in cardiac tissues (22–26). More recently, other groups similarly identified SK4 channels in the canine heart (27–29). These SK4 K^+^ channels are well known to be expressed by immune cells such as T cells, B cells, and macrophages, as well as by endothelial cells, fibroblasts, and proliferating smooth muscle cells of the vascular system (30–33). Recently, we demonstrated that the SK4 channel protein is widely expressed in the atria of rat and human hearts and to a lesser extent in the ventricles (23). A novel allosteric blocker, BA6b9, was designed to interact with the calmodulin-PIP2 binding domain, a previously untargeted region of these SK4 channels, at the specific interface of the proximal C-terminus and the linker S4–S5 domain (23). BA6b9 does not interact with the SK1, SK2, or SK3 K^+^ channels or with any other important cardiac channels. When used for the ex vivo treatment of rat hearts, BA6b9 as well as the generic SK4 channel blocker Tram-34 significantly prolonged atrial and atrioventricular effective refractory periods (AERP and AVERP, respectively) and inhibited the ability of carbachol to sustain AF following burst pacing. SK4 K^+^ channels are also expressed in mononuclear cells and fibroblasts (34), and their suppression by Tram-34 was recently shown to inhibit proinflammatory processes and tachypacing-related atrial remodeling in a canine model (27, 28). Thus, we hypothesized that in addition to the direct effect of SK4 K^+^ channel blockade on the atrial electrophysiology, it may have profound effects on atrial remodeling by inhibiting inflammation and fibrosis, which appear to be prominent in the context of HF (9, 35). Hence, we posited that SK4 K^+^ channels are proarrhythmic and proinflammatory such that blocking them may prevent atrial remodeling and AF progression in HF patients.

**Fig. 1.**
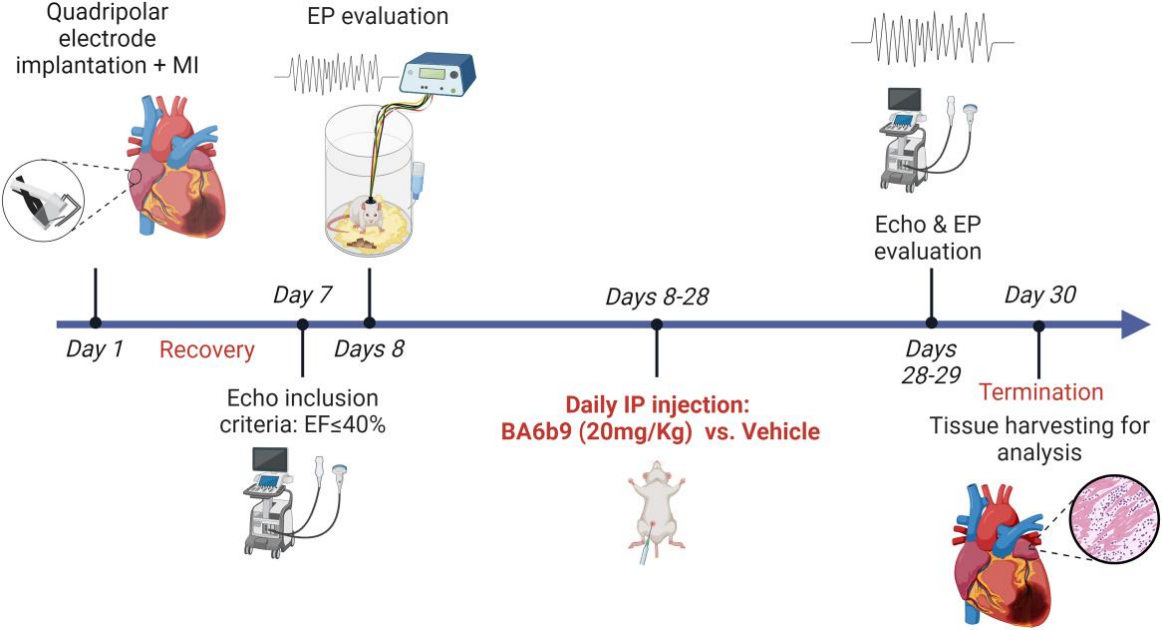
Experimental protocol and timeline for the assessment of the effects of BA6b9 treatment on the AF substrate of HFrEF rats post-MI. The initial operation (day 1) included left coronary artery ligation + quadrupolar electrode implantation for long-term EP evaluation. Rats with an EF ≤ 40% at 7 days post-surgery were randomly assigned to receive daily injections of either BA6b9 or vehicle for 3 weeks. At the end of treatment, final echocardiography and EP studies were performed and hearts were collected for further analysis. Created with Biorender.com.

In this study, we examined the ability of the SK4 channel allosteric blocker, BA6b9, to alter AF susceptibility and atrial remodeling in an HFrEF rat model (EF < 40%). The results of these analyses indicated that daily BA6b9 injections (20 mg/kg/day) for 3 weeks (Fig. 1) starting 1 week post myocardial infarction (MI) did not affect ventricular function but markedly attenuated both AF induction and duration in this setting. In addition, we found that the atrial levels of SK4 K^+^ channel expression were markedly upregulated in these rats post-MI in conjunction with the increased expression of NLRP3, the lateralization of the atrial connexin Cx43, and increased collagen deposition. Treatment with BA6b9 attenuated all of these detrimental changes, thus indicating that such drug-mediated blockade of SK4 K^+^ channels not only favors rhythm control but can also inhibit structural remodeling, which is a highly desirable effect, particularly in the setting of HF.

## Results

### In vitro confirmation of BA6b9 potency

**Fig. 2.**
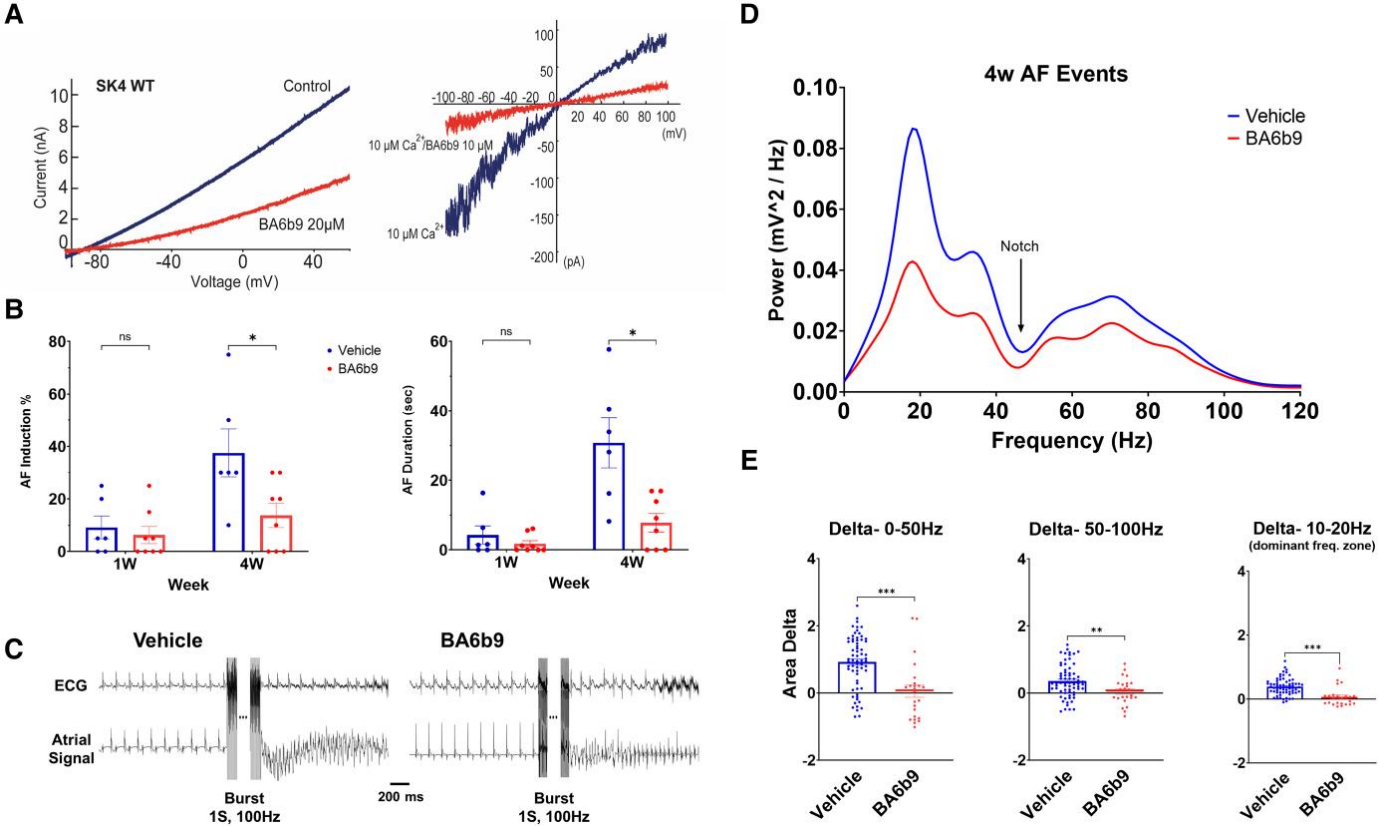
BA6b9 treatment reduces the AF substrate of post-MI rats. A) Confirmatory in vitro recordings. Left: Representative trace of WT SK4 currents in the absence and presence of 20 µM BA6b9, displaying a degree of inhibition of ≈56%. Right: Representative traces of an inside-out macropatch from a CHO cell expressing WT SK4 channels in the absence and presence of 10 µM BA6b9 under internal saturating calcium concentrations. Currents were recorded by 10 repetitive 1 s duration voltage ramps from −100 mV to +100 mV from a holding potential of 0 mV. B) BA6b9 treatment significantly reduced AF induction and AF duration. C) Example of postburst AF episodes in the vehicle (left) and BA6b9 (right) groups. D) Mean power spectrum of the AF episodes following long-term BA6b9 vs. vehicle treatment. Notably, the vehicle group demonstrated greater amplitudes over a wide range of frequencies relative to the BA6b9 group. Notch (arrow) indicates the zone where a notch filter was applied due to power supply-related noise (50 Hz). E) Quantitative analysis of delta between the power spectrum integrals of arrhythmic episodes and preburst NSR. Note the significantly higher values in the vehicle group compared to the AF events among BA6b9-treated rats.

Before performing the in vivo experiments, we confirmed that the batch of our BA6b9 drug worked properly on recombinant human SK4 K^+^ channels expressed in transfected Chinese hamster ovary (CHO) cells. As in our previous work, BA6b9 inhibited the SK4 K^+^ currents recorded using the whole-cell and the inside-out configurations of the patch clamp by 56 ± 2% (20 µM, *n* = 34, *P* < 0.0001) and 66 ± 5% (10 µM, *n* = 6, *P* = 0.0018), respectively (Fig. 2A).

### Long-term BA6b9 treatment attenuates the AF substrate of post-MI rats with HFrEF

Our instrumented electrophysiological (EP) device (36, 37), including the recently developed atrial-quadripolar electrode (38, 39), enabled a detailed evaluation of the impact of BA6b9 treatment on the supraventricular EP and particularly the AF substrate of post-MI rats in the freely moving state. The experimental design and flow of the current study are illustrated schematically in Fig. 1. As we previously demonstrated, the AF substrate that develops in this model is inversely correlated with EF (36). Thus, we specifically focused our current study on rats with an EF ≤ 40% at the end of the postoperative recovery week. In addition, since our study focused on atrial remodeling and AF substrate, we intentionally started the randomized treatment protocol only following this postoperative recovery week, so the acute recovery phase from device implantation and MI had already passed. As expected following randomization, the baseline parameters in the two arms of the study were not different from each other in terms of body weight, heart rate (HR), echocardiographic parameters (including EF), or various EP parameters (Table 1). Reliably predicting in vivo efficacy from in vitro data is very challenging, especially in the absence of quantitative pharmacokinetic/pharmacodynamics data for the BA6b9 compound. In a very preliminary approach and based on its in vitro efficacy (IC50 ≈ 8.5 µM), we decided to use BA6b9 at 20 mg/kg/day for 3 weeks. BA6b9 had no effect on body weight gain during the study. In addition, echocardiographic analysis revealed that BA6b9 treatment did not significantly affect LVIDd, LVIDs, EF, or LA diameter in these animals (Table 1). EP analysis indicated no effect of long-term BA6b9 treatment on the HR, the PR intervals, or the corrected sinus node recovery time (CSNRT). In line with our previous data showing that BA6b9 does not affect the cardiac IKs (Kv7.1 + KCNE1), Herg, Kv1.5 and Kv2.1 potassium current, BA6b9 did not alter the QT interval. Importantly, the BA6b9 treatment group did exhibit a significantly longer AERP at the end of the study as well as a trend towards an increase in AVERP that did not reach significance. Remarkably, BA6b9 treatment significantly reduced both AF induction and AF duration (Fig. 2B). In addition, eyeball tracing of arrhythmic episodes in the BA6b9 group typically indicated a less complex pattern (Fig. 2C). Accordingly, power spectrum analysis of the induced arrhythmic episodes revealed marked differences between the BA6b9- and vehicle-treated groups (Fig. 2D), with attenuated power in the BA6b9 group over a wide range of frequencies, which was most prominent around 15 Hz (the dominant frequency in both groups) (Figs. 2E and S1). Overall, these findings demonstrate that while BA6b9 did not alter ventricular function in HFrEF rats, it markedly attenuated the AF substrate, mildly increased AERP, and modified the properties of induced arrhythmic episodes.

**Table 1.** Characteristics of BA6b9-treated group vs. vehicle-treated group.

		BASE-1W			Final-4W			
		Vehicle	BA6b9	*P*-value	Vehicle	BA6b9	*P*-value	*n*
General	Weight	279.71 ± 22.3	264.25 ± 31.6	0.14	361 ± 14.9	344.5 ± 23.8	0.1384	7, 8
	HR	384.29 ± 27.7	362.63 ± 26.7	0.15	371 ± 62.8	386.5 ± 66.3	0.6512	7, 8
Echo	EF	32.77 ± 5.4	30.61 ± 6.8	0.51	33.67 ± 9	31.13 ± 13	0.667	7, 8
	LVIDd	8.42 ± 0.7	8.99 ± 0.5	0.07	9.42 ± 1.4	10.32 ± 0.6	0.11	7, 8
	LVIDs	6.42 ± 1.02	7.10 ± 0.64	0.46	7.69 ± 1.84	8.38 ± 1.21	0.69	7, 8
	LA	6.41 ± 0.4	6.76 ± 0.8	0.43	6.43 ± 0.8	6.96 ± 0.4	0.2468	5, 6
ECG/EP	PR	52.59 ± 3.51	50.71 ± 2.81	0.27	55.27 ± 2.79	53.51 ± 5.17	0.4372	7, 8
	QT	74.83 ± 15.9	83.91 ± 13	0.27	74.13 ± 8.2	77.34 ± 8.1	0.47	7, 7
	AERP 70CL	30.75 ± 8.4	40.17 ± 6	0.06	29.75 ± 3.4	37.67 ± 3.6	**0**.0095**	4, 6
	AERP 120CL	31.75 ± 7.8	39.33 ± 5.9	0.1	30 ± 4.2	37.33 ± 4.1	**0**.0286*	4, 6
	AVERP 120CL	67.4 ± 5.8	66.29 ± 3.5	0.73	69.33 ± 6	74.86 ± 2	0.1212	5, 7
	SNRT/SCL %	12% ± 6%	12% ± 2%	0.89	12% ± 3%	12% ± 3%	0.81	6, 8
	CSNRT	18.52 ± 8.9	17.53 ± 3	0.77	18.3 ± 7	19.38 ± 5.3	0.75	6, 8

HR, heart rate (BPM); EF, ejection fraction (%); LVIDd, left ventricular internal diameter-diastolic (mm); LVIDd, left ventricular internal diameter-systolic (mm); LA, left-atrial diameter (mm); AERP, atrial effective refractory period; AVERF, AV node refractory period; AERP 70 CL, AERP obtained with 70 ms basic cycle length (ms); AERP 120 CL, AERP obtained with 120 ms basic cycle length (ms); AVERP 120 CL, AVERP obtained with 120 ms basic cycle length (ms).

Bold text indicates statistical significance. *P < 0.05. **P < 0.01.

### BA6b9 inhibits the post-MI structural remodeling of the LA

As SK4 K^+^ channels are not only expressed in cardiomyocytes but also in fibroblasts and macrophages (23, 28–30, 32, 33), which all possess the inflammatory signaling machinery (17), we hypothesized that their pharmacological inhibition can reduce AF substrate by preventing atrial structural remodeling.

**Fig. 3.**
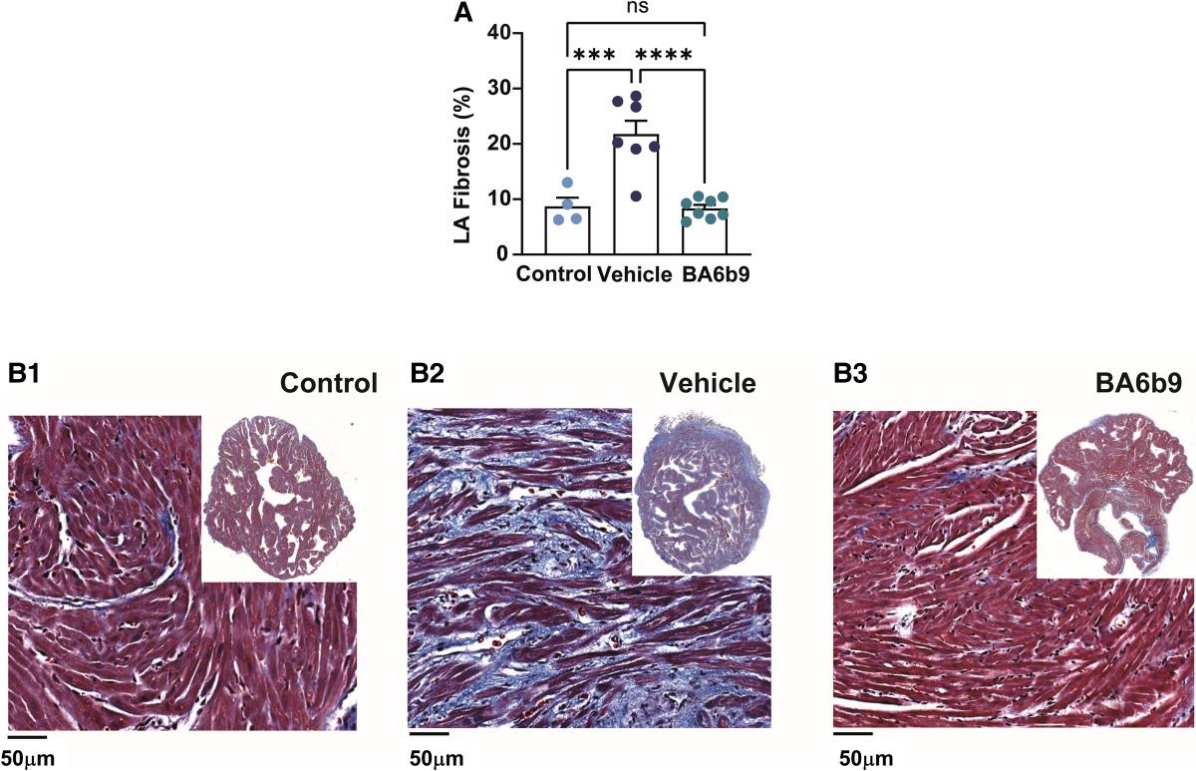
Effect of BA6b9 on collagen deposition in the left atrium of rats with MI-induced HF. A) Statistical summary of total left-atrial fibrosis; vehicle- vs. BA6b9-treated rats, compared to control (*n* = 7, *n* = 8, *n* = 4, respectively; one-way ANOVA, F(2, 16) = 16.53, Sidak's multiple comparisons test *P* = 0.0001). B1) Representative Masson's trichrome-stained histological LA cross-section from a control rat (left). B2, B3) Representative Masson's trichrome-stained histological cross-sections of the LA from post-MI rats treated with vehicle (middle) or BA6b9 (right) for 21 days. Blue staining indicates collagen deposition. An inset in each photograph shows the full LA tissue in low resolution.

In order to properly evaluate the structural effects of BA6b9 treatment in the post-MI setting, we added a control group of naïve rats to the analysis. As expected, Fig. 3 shows that collagen deposition in the LA as measured by Masson's trichrome staining markedly increased in vehicle-treated rats subjected to MI as compared to control rats. In contrast, BA6b9 treatment for 3 weeks markedly attenuated LA collagen deposition in the post-MI setting. Along the same line, the expression of α-SMA, a marker that is known to increase under cardiac stress conditions such as pathological hypertrophy (40), was also increased in the LA of vehicle-treated rats and was markedly inhibited by BA6b9 treatment (Fig. S2). In addition, staining with Wheat Germ Agglutinin (WGA), which binds and labels glycoproteins of the cell membrane as well as extracellular fibrotic tissue (41), was markedly increased in the LA of post-MI rats relative to the controls and was markedly inhibited by BA6b9 treatment in post-MI rats (Fig. S3). Overall, our findings highlight the remarkable ability of BA6b9 to abrogate the atrial structural remodeling induced by HFrEF in the post-MI setting.

### BA6b9 treatment reduces the upregulation of atrial SK4 K^+^ channels and NLRP3 following MI

**Fig. 4.**
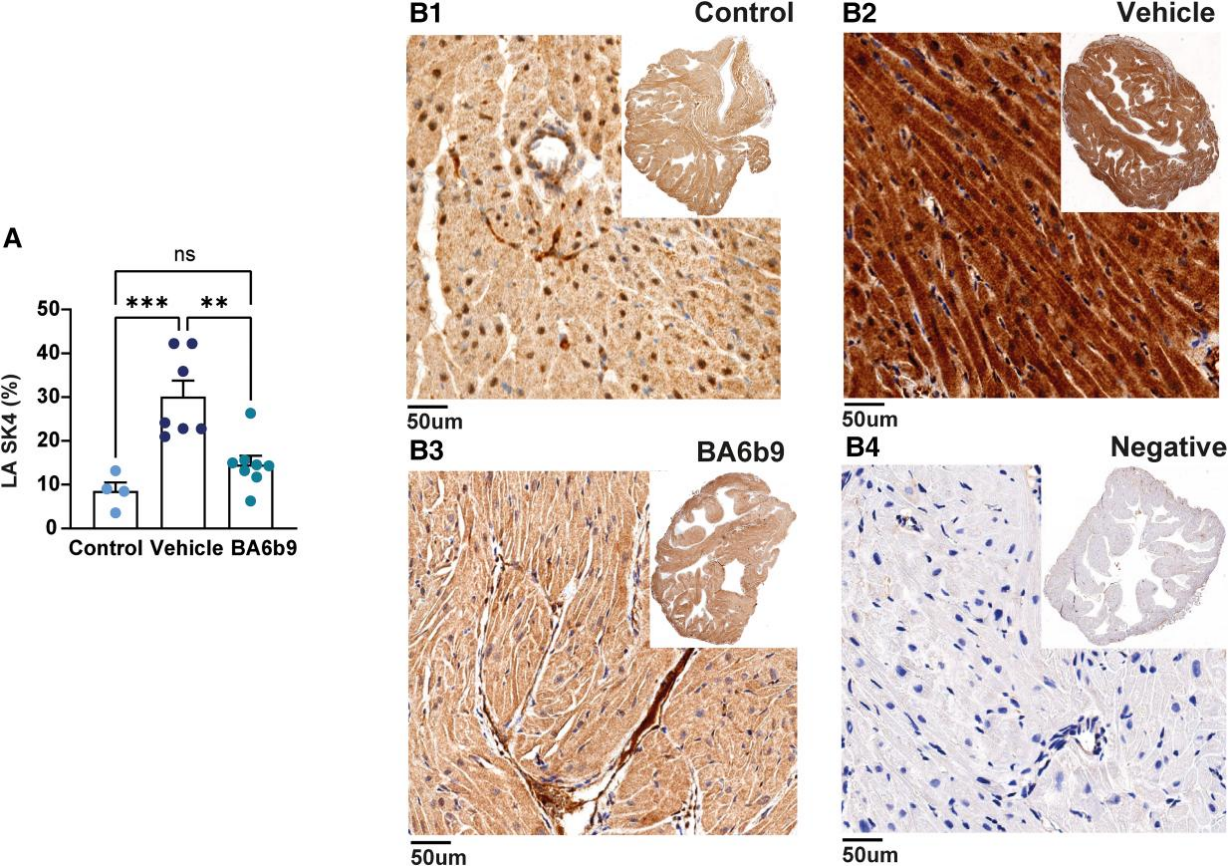
Effect of BA6b9 on SK4 expression in the left atrium of rats with MI-induced HF. A) Statistical summary of overall left-atrial SK4 expression; vehicle- vs. BA6b9-treated rats, compared to control (*n* = 7, *n* = 8, *n* = 4, respectively; one-way ANOVA, F(2, 16) = 14.27, Sidak's multiple comparisons test *P* = 0.0003). B1) Representative histological LA cross-section from a control rat (upper left), stained with DAB. B2, B3) Representative DAB-stained histological cross-sections of the LA from post-MI rats treated with vehicle (upper right) or BA6b9 (lower left) for 21 days. Brown staining intensity indicates the level of SK4 expression in the tissue, with darker brown indicating stronger expression. B4) Negative control SK4 staining. An inset in each photograph shows the full LA tissue in low resolution.

**Fig. 5.**
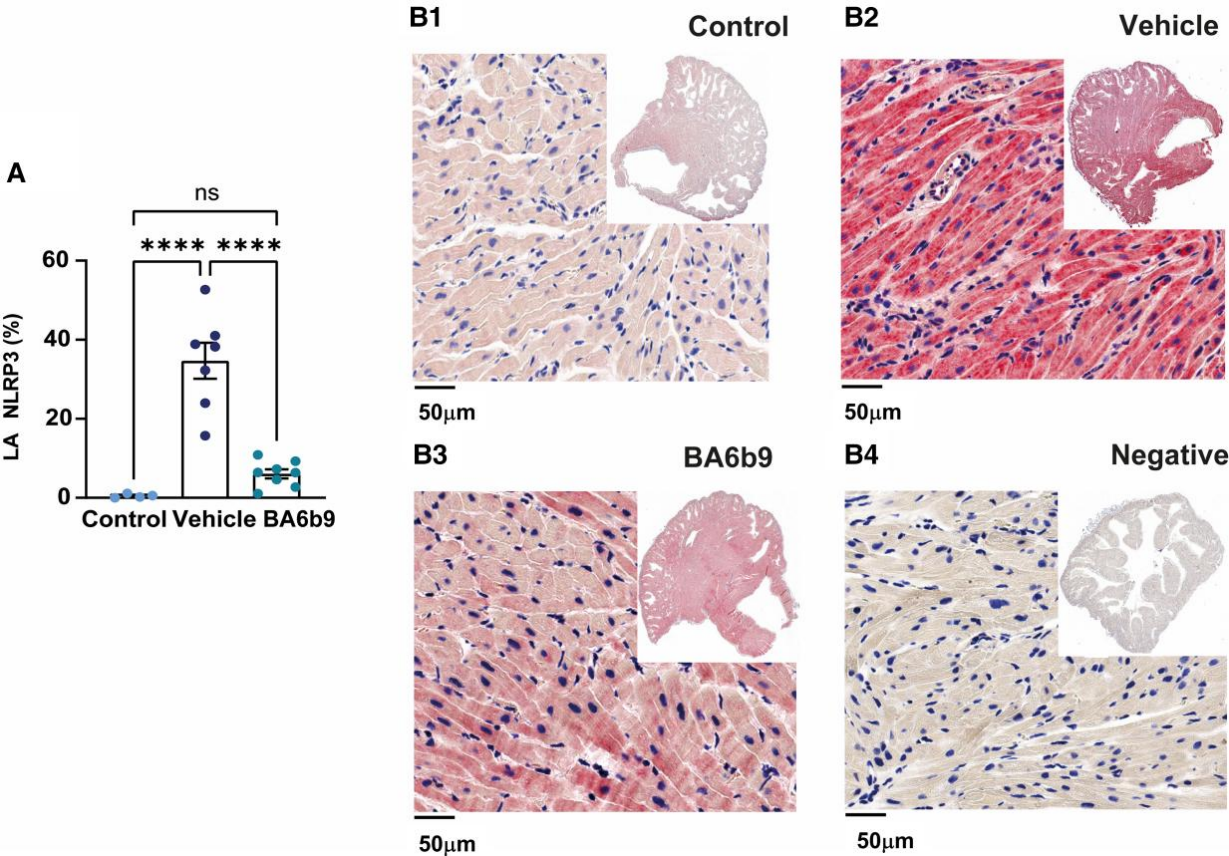
Effect of BA6b9 on NLP3 expression in the left atrium of rats with MI-induced HF. A) Statistical summary of entire left-atrial NLRP3 expression; vehicle- vs. BA6b9-treated rats, compared to control (*n* = 7, *n* = 8, *n* = 4, respectively; one-way ANOVA, F(2, 16) = 35.38, Sidak's multiple comparisons test *P* < 0.0001). B1) Representative histological LA cross-section from a control rat (upper left), stained with Sirius Red. B2, B3) Representative Sirius Red-stained histological cross-sections of the LA from post-MI rats treated with vehicle (upper right) or BA6b9 (lower left) for 21 days. Positive red staining indicates NLRP3 expression. B4) Negative control NLRP3 staining. An inset in each photograph shows the full LA tissue in low resolution.

In line with our hypothesis that SK4 K^+^ channels are involved in atrial remodeling in the context of HF, we found that SK4 K^+^ channel expression in the LA, as measured by immunohistochemistry (IHC), was noticeably upregulated in vehicle-injected rats subjected to MI as compared to control rats (Fig. 4). Interestingly, post-MI blockade of SK4 K^+^ channels with BA6b9 markedly reduced this upregulation of atrial SK4 K^+^ channels in the LA, indicating that the overexpression of SK4 K^+^ channels is both a marker of the stressed atrial myocardium, as well as an active player in the stress that leads to atrial remodeling (see Discussion). The NLRP3 inflammasome is known to be activated during atrial remodeling and AF progression in animal models and patients with AF (17, 42, 43). As shown in Fig. 5, in the LA, NLRP3 expression measured by IHC was markedly increased in vehicle-treated rats subjected to MI as compared to control rats, while BA6b9 treatment virtually eliminated NLRP3 upregulation in this setting.

### BA6b9 reduces the lateralization of atrial Cx43 in post-MI rats

**Fig. 6.**
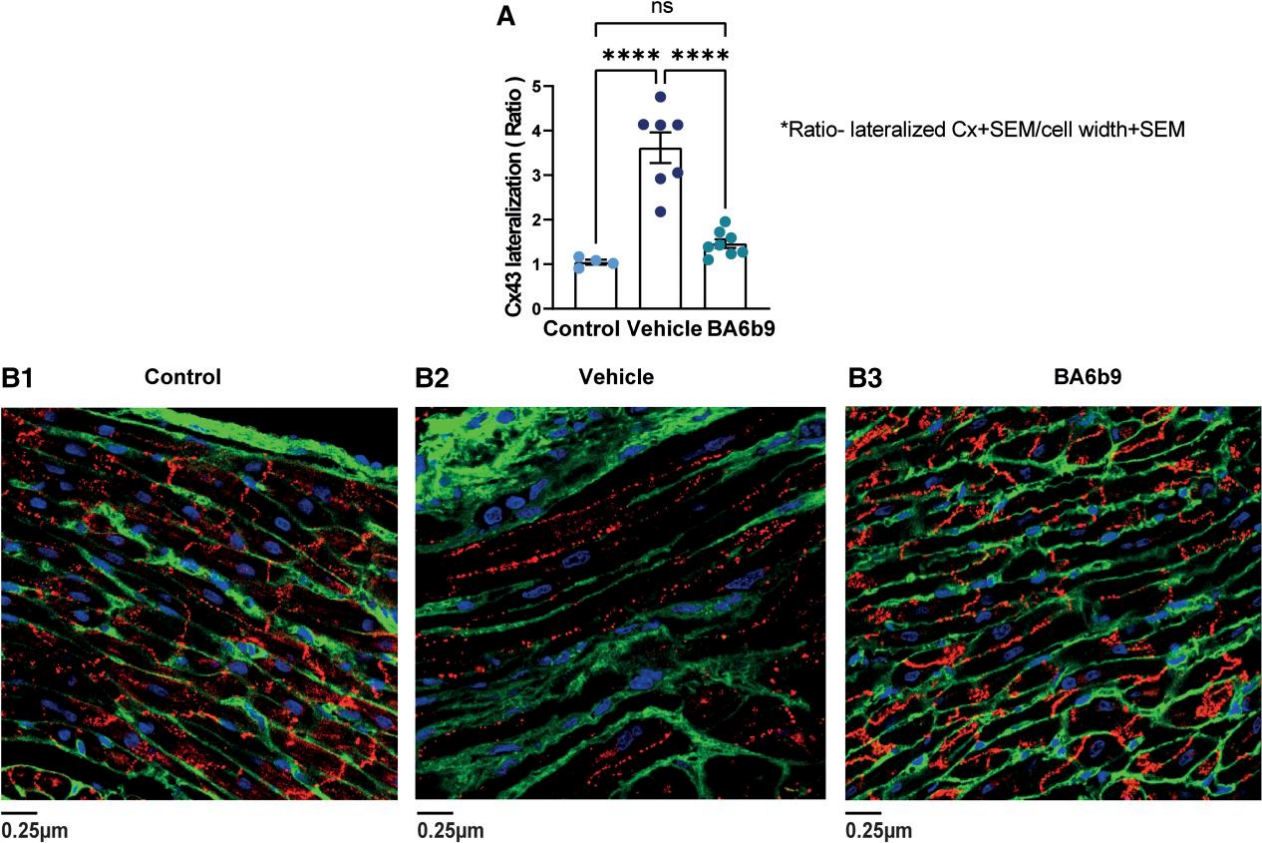
Effect of BA6b9 on Cx43 distribution in the atrial myocardium of rats with MI-induced HF. A) Statistical summary of Cx43 lateralization in the left-atrial myocardium (analysis of 200 atrial cardiomyocytes in longitudinal sections, per animal). Cx43 distribution was analyzed in immunofluorescent confocal images stained for Cx43 (red), cardiomyocyte membranes (green), and nuclei (blue). Cx43 lateralization was determined to be evident only if the red Cx43 staining paralleled/colocalized with the longitudinal cardiomyocyte membranal staining in green. Lateralization is presented and quantified as the ratio between Cx43 length (µm) when paralleled/colocalized with the cell membrane to the cell's width. The histogram compares values for control, vehicle-treated, and BA6b9-treated rats (*n* = 4, *n* = 7, *n* = 8, respectively; one-way ANOVA, F(2, 16) = 34.22, Sidak's multiple comparisons test *P* < 0.0001). B1) Representative longitudinal section of the LA from a control rat (left), demonstrating clear and strong Cx43 expression in the intercalated discs. B2) Representative longitudinal section of the LA from post-MI rats treated with vehicle (middle). Note the marked lateralization of Cx43 as well as the evident reduction in Cx43 expression and increased cellular hypertrophy compared to control. B3) Representative longitudinal section of the LA from post-MI rats treated with BA6b9 (right) for 21 days. Note that Cx43 intensity and distribution in the intercalated discs are similar to those in control rat sections (significant decrease in Cx43 lateralization) with a corresponding reduction in cellular hypertrophy.

Remodeling of Cx43 is commonly observed in patients with AF (44). The shift of connexins from the intercalated disc to the lateral membranes of cardiomyocytes is referred to as lateralization and can slow the conduction velocity and increase conduction heterogeneity (44). Thus, we conducted immunofluorescence experiments to assess whether the AF substrate of post-MI rats is associated with altered atrial localization of Cx43 and, if so, whether the blockade of SK4 K^+^ channels could inhibit this lateralization process. Indeed, we found that the lateralization ratio of Cx43 markedly increased following MI, while BA6b9 treatment could potently inhibit this remodeling process (Fig. 6).

### BA6b9 treatment inhibits post-MI structural changes of the LA epicardium

**Fig. 7.**
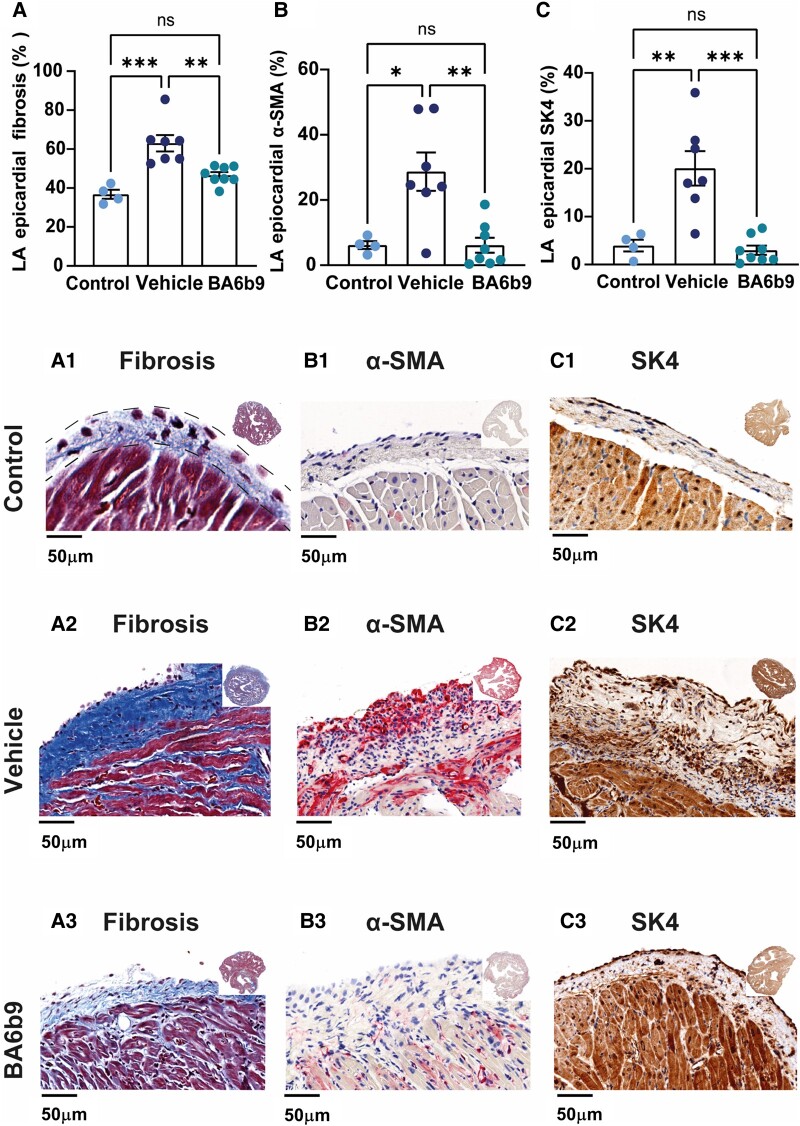
Effect of BA6b9 on collagen deposition, α-SMA expression, and SK4 expression in the LA epicardium of rats with MI-induced HF. A) Statistical summary of LA epicardial fibrosis (analysis of six randomized epicardial fields for each atrial section, total of 18 atrial-epicardium fields per animal); vehicle- vs. BA6b9-treated rats, compared to control (*n* = 7, *n* = 8, *n* = 4, respectively; one-way ANOVA, F(2, 16) = 16.53, Sidak's multiple comparisons test *P* = 0.0001). A1) Representative histological cross-section of LA myocardium attached to the epicardial tissue from a control rat (left upper row). The analyzed area is marked by dashed lines. A2, A3) Representative histological cross-sections of the LA from post-MI rats treated with vehicle (left middle row) or BA6b9 (left lower row) for 21 days. Sections A1–A3 were stained with Masson's Trichrome. B) Statistical summary of LA epicardial α-SMA expression; (analysis: same as in A; vehicle vs. BA6b9-treated rats, compared to control (*n* = 7, *n* = 8, *n* = 4, respectively; one-way ANOVA, F(2, 16) = 16.53, Sidak's multiple comparisons test *P* = 0.0001). B1) Representative histological cross-section of LA myocardium attached to the epicardial tissue from a control rat (middle upper row). B2, B3) Representative histological cross-sections of the LA in post-MI rats treated with vehicle (center) vs. BA6b9 (middle lower row) for 21 days. Sections B1–B3 were stained with Sirius Red. C) Statistical summary of LA epicardial α-SMA expression; (analysis: same as in A; vehicle- vs. BA6b9-treated rats, compared to control (*n* = 7, *n* = 8, *n* = 4, respectively; one-way ANOVA, F(2, 16) = 10.21, Sidak's multiple comparisons test *P* = 0.0014). C1) Representative histological cross-section of LA myocardium attached to the epicardial tissue from a control rat (right upper row). C2, C3) Representative histological cross-sections of the LA in post-MI rats treated with vehicle (right middle row) or BA6b9 (right lower row) for 21 days. Sections C1–C3 were stained with DAB. Note the marked thickening of the atrial epicardium in MI rats compared to controls as well as the significant reductions in collagen deposition (A, A1–A3), α-SMA expression (B, B1–B3), and SK4 expression (C, C1–C3) in the BA6b9-treated rats compared to the vehicle group. An inset in each photograph shows the full LA tissue in low resolution.

Data from recent years support the notion that the atrial epicardium plays important roles in atrial electrical and structural remodeling. AF is accompanied by progressive epicardial fibrosis affecting transmural conduction (45–48). In addition, the secretory epicardial adipose tissue enfolding the atria secretes various proinflammatory and profibrotic mediators that promote atrial remodeling and increase susceptibility to AF (49). While the atrial epicardium is far less studied in rodent models, we have noted marked changes in the LA epicardium of post-MI rats relative to controls. Therefore, we aimed to systematically examine these changes as well as the effects of SK4 channel blockade by BA6b9 on the LA epicardial tissue. Indeed, LA epicardial fibrosis measured by Masson's trichrome staining was significantly increased in vehicle-injected rats subjected to MI as compared to control rats, while BA6b9 treatment inhibited the development of excess epicardial fibrosis (Figs. 7A and A1–A3). Very similar results were obtained when α-SMA expression was quantified (Figs. 7B and B1–B3). The LA epicardial remodeling post-MI was also characterized by the upregulation of SK4 K^+^ channels that was potently blocked by BA6b9 (Figs. 7C and C1–C3), and a similar pattern was noted with respect to the expression of the NLRP3 inflammasome (Fig. S4).

## Discussion

Based on the initial assumption that SK4 K^+^ channels are proarrhythmic and proinflammatory, in this study, we examined whether atrial SK4 channels are upregulated in the post-MI setting in rats and whether their blockade using our recently developed allosteric inhibitor BA6b9 could mitigate the AF substrate and atrial remodeling that develop in this HFrEF model, which mimics important aspects of MI and AF comorbidity in humans (36, 50, 51).

As previously shown, post-MI rats develop a progressive increase in their AF substrate over time that correlates with the level of systolic dysfunction. In addition, there is a clear association between atrial fibrosis and the sustainability of arrhythmic events (36). In humans, HF occurs as frequently as AF, and the coexistence of the two diseases is very common, leading to poor clinical outcomes (9, 10). HfrEF, in particular, stimulates ion channel remodeling, inflammation, and interstitial fibrosis, which makes the atria more prone to AF (1, 17). Thus, the HFrEF rat model of post-MI AF substrate development is clinically relevant. In addition, technical advances introduced by our group (38, 39) enabled us to chronically implant an atrial-quadripolar electrode for simultaneous pacing and high-resolution recordings in unanesthetized rats, making this model even more potent and valuable for atrial EP and AF substrate analyses.

In this work, we demonstrated that in vivo BA6b9 treatment for 3 weeks, starting 1 week following LAD ligation, prolongs the AERP, reduces AF induction and duration, and markedly affects the power spectrum of induced AF episodes. Importantly, our final EP analysis was performed ∼24 h following the last injection of BA6b9. This design was intended to allow the animals to acclimate to the EP cage overnight before EP testing in the resting state. However, it also enabled us to focus on the sustained effects of the treatment rather than the acute effects of BA6b9. We recently demonstrated in the isolated rat hearts ex vivo that both Tram-34 and BA6b9 were able to significantly prolong the RR and PR intervals and to increase the AERP and the AVERP without affecting the ventricular effective refractory period (23). Prolongation of RR interval (i.e. bradycardia) and PR interval were also noted in unanesthetized mice following acute applications of SK4 channel blockers (24). Thus, the fact that in our final EP study the RR, PR, and AVERP were not significantly different between the BA6b9-treated and the vehicle-treated rats clearly supports the notion that the main effects we detected here are indeed sustained. Another important aspect of our study design was the delayed initiation of treatment on day 7 following MI induction. This was intentionally performed to avoid possible effects on acute postoperative recovery. However, since our main focus was on atrial remodeling, it was also important to avoid possible effects of SK4 channel blockade on the acute inflammatory phase of post-MI healing (52, 53). Indeed, while our data indicate that the BA6b9 treatment was well tolerated and did not affect the weight gain in experimental rats, it also did not affect ventricular function using our current treatment protocol. This issue is critical since it strongly supports the notion that the inhibition of AF substrate and atrial remodeling we describe here are related to the effects of BA6b9 on the atria rather than to indirect effects on ventricular function. Of note, given the existing data regarding the possible role of SK4 channels in the acute inflammatory phase of post-MI healing (52, 53), it would be interesting to test the effects of early post-MI treatment with BA6b9 on ventricular healing and remodeling. However, this issue was out of the scope of the present study.

Our histological findings indicate that blocking SK4 K^+^ channels with BA6b9 not only attenuates AF induction and duration but also dramatically reduces post-MI overexpression of the SK4 channels themselves as well as multiple measures of atrial structural remodeling. Studies in recent years have revealed a key role for inflammatory signaling pathways in AF development (17). Inflammation is also recognized as an important pathophysiological mediator in the post-MI HFrEF setting (54). Though inflammatory signaling crucially involves noncardiomyocyte cells such as fibroblasts, adipocytes, or macrophages, emerging evidence indicates that atrial cardiomyocytes have the same inflammatory signaling pathways, the activation of which contributes to AF substrate development (17, 55–57). Indeed, atrial cardiomyocytes were not only shown to secrete proinflammatory cytokines but also to express their respective receptors that can stimulate various important AF-related signaling cascades via autocrine or paracrine mechanisms (17). Although our work did not explore which specific signaling pathway may be involved, we found that 3 weeks of SK4 K^+^ channel blockade by treatment with BA6b9 drastically prevented atrial structural remodeling, as seen by the inhibition of increased collagen deposition (Masson's trichrome staining), extracellular matrix (ECM) accumulation (fluorescently conjugated WGA), and α-SMA expression when comparing BA6b9- and vehicle-treated post-MI animals. BA6b9 treatment also inhibited the upregulation of NLRP3 inflammasome in the LA of post-MI rats. Recent evidence demonstrates that NLRP3 inflammasome activation in atrial cardiomyocytes may be a necessary and sufficient condition for AF occurrence (17). In addition, NLRP3 inflammasome activity is increased in atrial cardiomyocytes of patients with paroxysmal AF or long-lasting persistent AF (17). Importantly, increases in cytosolic Ca^2+^ levels can lead to mitochondrial Ca^2+^ overload and the generation of mitochondria-derived reactive oxygen species, with subsequent NLRP3 inflammasome activation (58, 59). This latter feature might provide an additional deleterious link between abnormal diastolic Ca^2+^ handling, NLRP3 activation, and increased SK4 channel activity. Reflecting their probable proinflammatory nature, the atrial SK4 K^+^ channels were upregulated by 3.5-fold in the setting of HFrEF post-MI. Remarkably, the blockade of the SK4 K+ channels by BA6b9 treatment virtually suppressed this upregulation of atrial SK4 channels as well as that of the NLRP3 inflammasome.

The profound effects of BA6b9 treatment on collagen accumulation and the upregulation of α-SMA, the SK4 channel, and NLRP3 in the atrial epicardium also strengthens the clinical relevance of targeting AF by blocking SK4 K^+^ channel activity. Indeed, various studies support the view that endo-epicardial dissociation is an important driving force for breakthroughs to occur in AF. Moreover, in large mammals, epicardial adipose tissue properties correlate with AF severity, postoperative AF incidence, and AF recurrence rates after cardioversion or ablation (60). Although rodents do not normally have notable epicardial adipose tissue, it will be important to further evaluate how the noted above epicardial changes can contribute to the post-MI AF substrate and whether BA6b9 can have similar effects on the epicardium in large mammalian models.

AF progression also entails connexin remodeling. Hence, a progressive reduction in Cx43 levels, often accompanied by enhanced lateralization, has been described in myocardial samples from patients with HF of different etiologies, including those secondary to AF, dilated, and inflammatory cardiomyopathy (44, 61). The topological shift of Cx43 from the intercalated disc to the lateral membranes of cardiomyocytes is often accompanied by changes in phosphorylation and ischemia, including the progressive dephosphorylation of Cx43. The mislocalization of connexins outside intercalated discs and modifications in their phosphorylation status have been shown to result in important alterations of cardiomyocyte electrical coupling, thus disturbing action potential propagation and contributing to arrhythmia susceptibility (44). In this work, BA6b9 treatment largely reversed the lateralization of the atrial connexin Cx43 in this post-MI rat model of HF.

At this stage, it is difficult to conclude which of the BA6b9-dependent effects (i.e. AERP prolongation, inhibition of atrial inflammation/fibrosis, and attenuation of Cx43 lateralization) contribute to the potent AF substrate inhibition mediated by this treatment. However, all of these findings strongly support the notion that such treatment can have multiple beneficial long-term effects that are independent of the purely EP effects we have recently described (23). Current pharmacological therapies aiming to convert AF to sinus rhythm (“rhythm control” modalities) are very limited, especially in the setting of HF (1, 12–14, 16, 30, 62–66). Moreover, none of these therapies have been shown to prevent the progressive structural changes such as inflammation and interstitial fibrosis that appear to be critical for AF progression. In this regard, our novel therapeutic strategy aimed at targeting the deleterious overexpression of the atrial SK4 K^+^ channels is a novel approach that appears to address both atrial electrical and structural remodeling in a favorable manner.

## Limitations

Our study does not provide information about which cell type is specifically affected by SK4 channel blockade, and it is likely that complex multidirectional autocrine and paracrine signaling pathways are involved in this process. However, precisely because of their unique expression in all cell types that are involved in AF induction and progression, SK4 K^+^ channels represent a suitable new target for drugs that can combine the advantages of rhythm control and antiremodeling therapy. In addition, while our immunohistochemical analysis of SK4 K^+^ channels clearly indicates that the atrial overexpression of these channels is at least partially related to cardiomyocytes, we were not able to reliably determine the exact cellular localization of the overexpressed channels. Nevertheless, our clear evidence regarding the beneficial effects of BA6b9 demonstrates that this SK4 K^+^ channel overexpression is a critical aspect of the pathophysiology in this setting rather than an epiphenomenon. Finally, our current proof-of-concept study was performed using only male rats. The logic for this design is that the AF substrate of freely moving female rats was found to be substantially lower compared to male rats of the same age, in an experiment we performed (data not shown). Thus, similar conclusive results for female rates have yet to be generated.

## Materials and methods

### Patch clamp validation recordings

The in vitro expression of SK4 channels and confirmatory whole-cell and inside-out patch clamp recordings were performed as described in detail in Burg et al. (23). Briefly, human SK4 inserted into the pEGFP-C1 vector (pEGFP-SK4) was used to transfect CHO cells that were grown in Dulbecco's modified Eagle's medium supplemented with 2 mM glutamine, 10% fetal calf serum, and antibiotics, The cells were seeded (20,000/well) on poly-L-lysine-coated glass coverslips (13 mm in diameter) in a 24-multiwell plate and transfected with 0.5 µg pEGFP-SK4 using the TransIT-LT1 Transfection Reagent (Mirus Bio) according to the manufacturer's protocol. For electrophysiology, transfected cells were visualized approximately 40 h after transfection with a Zeiss Axiovert 35 inverted fluorescence microscope.

### BA6b9

BA6b9 synthesis has been described in detail previously (23). For in vitro validation studies, BA6b9 powder was dissolved in DMSO and applied to the cells at a concentration of 10 μM. The final concentration of DMSO was 0.1% (23). For rat studies, BA6b9 was dissolved in sesame oil from Sigma-Aldrich (St. Louis, MO, USA) to create a stock solution of 10 mg/mL which was stored at 4°C and injected intraperitoneally once per day at a concentration of 20 mg/kg. Vehicle treatment consisted of an equivalent volume of sesame oil alone.

### Animals

All animal experiments in this study were approved by the Institutional Ethics Committee of Ben-Gurion University of the Negev, Israel (Protocol IL-24-05-2020D). Experiments were performed on adult (8 weeks old, ∼280 g) male Sprague-Dawley (SD) rats obtained from Envigo Laboratories LTD (Jerusalem, Israel). Rats were kept under standardized conditions throughout the study (36, 39). The rats had environmental enrichment and were monitored on a daily basis for signs of stress or inappropriate weight loss, according to guidance from Ben-Gurion University veterinary services (assured by the Office of Laboratory Animal Welfare [OWLA] no. A5060-01) and fully accredited by the Association for Assessment and Accreditation of Laboratory Animal Care International (AAALAC).

### Implantable EP devices

For atrial EP studies we used a chronically implanted EP device, which has previously been described in detail (36, 38, 39) and includes peripheral ECG leads as well as an atrial quadripolar electrode adapted for advanced supraventricular EP studies in the freely moving state. Briefly, the device includes an 8-pin connector that is attached by highly flexible insulated electrical wires (AS155-36, Cooner wires, Chatsworth, CA, USA) to the atrial-quadripolar electrode and to three additional peripheral ECG leads. The atrial-quadripolar electrode is composed of 4 Platinum–Iridium electrical poles that are embedded in medical grade silicon (MED-6219P, Nusil, CA, USA) and fixed to the atria by miniature stainless steel hooking pins (26002-10, Fine Science Tools, Vancouver, Canada). Full details and photos of the design and fabrication of the quadripolar electrode are described in Murninkas et al. (39). All components of the instrumented device are biocompatible. Following preparation and before instrumentation, each device was thoroughly cleaned with 70% ethanol and sterilized by electron beam radiation.

## Surgical procedure for EP device implantation and induction of MI

A procedure including EP device implantation and left coronary artery ligation was performed in each animal as previously described (36). Briefly, animals were anesthetized (intramuscular ketamine/xylazine 75/5 mg/kg), mechanically ventilated, and placed on a heating pad. Under sterile conditions, left thoracotomy was performed and the left coronary artery was ligated followed by chest closure. Next, right thoracotomy was performed and the atrial-quadripolar electrode was placed on the right atrium followed by chest closure. ECG leads were inserted subcutaneously, and the device connector was exteriorized through the skin of the neck. A shielding ring that was sutured to the skin of the neck prevented extraction of the device over time (36). Postoperative recovery and analgesia were performed as described previously (36, 39).

## Echocardiography

Echocardiography measurements were performed with the Vevo 3100 Preclinical Imaging System (FUJIFILM VisualSonics, Canada) as previously described (36, 39). During the procedure, rats were lightly anesthetized with 1.5% isoflurane and positioned on a heating pad to maintain a rectal temperature of ∼37°C. 2D images of the left ventricle were taken on the parasternal long- and short-axis, and apical four-chamber views were recorded. Long- and short-axis M-mode images were obtained at the mid-papillary muscle area. LV end-diastolic diameter (LVIDd) and LV end-systolic diameter (LVIDs) were obtained from the long-axis M-mode trace. Left ventricular EF was calculated using planimetry as follows: EF = 100 × (LVIDd − LVIDs/LVIDd). LV trace was obtained from parasternal long-axis B-Mode. Left-atrial diameter was obtained from an apical four-chamber view. All measurements were averaged for three consecutive cardiac cycles and made by an experienced technician who was blinded to the pharmacological treatment of the animals.

## Experimental design and EP evaluation

Seven days following the initial surgery the first echocardiogram was performed. Rats having an LV trace EF ≤ 40% were considered as having significant LV dysfunction and were included in the study and connected to the EP setup for the initial EP study on the next day (postoperative day 7). Following this initial EP study, animals were randomized for treatment with daily intraperitoneal (i.p.) injections of either BA6b9 (20 mg/kg/day) dissolved in sesame oil or vehicle treatment (sesame oil alone). On postoperative day 28, the animals went through a final echocardiography scan and were connected to the EP setup for a final EP study on the next day. This EP study was performed 20–24 h following the last i.p. injection of BA6b9 or vehicle. Following the final EP study, tissues were collected under deep pentobarbital anesthesia.

EP studies were performed as previously described (39), with minor modifications. Briefly, for each EP evaluation, rats were placed in dedicated recording chambers where each instrumented device connector was attached to the pacing and recording apparatus via an elastic electrical cable. The proximal part of the elastic cable was connected to the EP system through a multichannel commutator (PLA-SL12C/SB, PLASTICS One Inc., CA, USA) that maintained stable electrical connections while the rats were freely rotating in the cages. In each animal, two atrial poles were selected for pacing and were electrically connected to an optically isolated pacing unit (STG4002-16 mA, Multichannels, Reutlingen, Germany). The remaining two atrial poles and the three peripheral ECG electrodes were connected to a voltage amplifier (Amplifier 1700, A-M systems, Carlsborg, WA, USA). As previously described (39), the electrode side that was used for pacing was empirically determined based on a relatively low capture threshold and an ability to differentiate the atrial signal from the stimulus artifact in the recordings from the other side. Signals were filtered (1–1,000 Hz) and sampled to the PC at a digital sample rate of 2 kHz. A self-made program created using Labview 7.1 (National Instruments, Austin, TX, USA) controlled data acquisition and electrical stimulation. EP studies were performed in an unanesthetized state following overnight adaptation to the EP cages and during daylight hours of the circadian cycle when animals were inactive, as previously described (36, 37, 39). RR, PR, and QT intervals were averaged from five cycles on the nonpaced ECG. An atrial pacing capture threshold was obtained using bipolar square current pulses (total duration 4 ms; 2 ms in each direction), and stimulus intensity was raised to double the threshold for the rest of the EP study. A programmed S1S2 stimulation protocol (S1 = 10) was used to determine the atrioventricular node refractory period (AVERP) and the atrial effective refractory period (AERP) in the millisecond range. AVERP was determined using a basic cycle length (BCL) of 120 ms. AERP measurements were performed using two different basic BCL: 70 and 120 ms. AVERP and AERP were confirmed three consecutive times. Sinus node recovery time (SNRT) was obtained using 30 s burst pacing protocols applied 3 times with a 30 s pause between the bursts. Arrhythmic substrate evaluation included 20 consecutive triggering bursts (1 s duration, 10 ms cycle length). Arrhythmic episodes lasting more than 4 min were aborted using short (1 s) pacing bursts of increasing intensity until sinus rhythm was restored. The minimal time between pacing bursts was 1 min from the end of an event. If an episode of more than 60 s was detected, the delay from the end of this episode to the next pacing burst was equal to the duration of the AF episode. The cutoff for defining a positive arrhythmic event was defined as >1 s following the burst pacing protocol.

To avoid any bias in the AF analysis by regular stable arrhythmic episodes (36, 39), we distinguished between regular and irregular events in our analysis. We have previously noted that regular arrhythmic episodes are characterized by a stable cycle length >60 ms (38). Thus, AF was defined in the current study as fast irregular atrial electrocardiograms or atrial waveforms in which the main repeating component had a duration <55 ms. Regular arrhythmic waveforms were analyzed separately from the AF analysis.

## Power spectrum analysis of AF waveforms

For power spectrum analysis of the AF waveforms, we cleaned the atrial signals from ventricular mixing based on QRS detection in the peripheral ECG, as described in detail in Murninkas et al. (38). Next, power spectrum analysis of the AF signals was performed using conventional Fast Fourier Transform (38). Since recordings were all performed in the presence of a notch filter to reduce the signal levels proximal to 50 Hz, an artificial depression was detected around this frequency (38).

## Histology

Hearts were excised and thoroughly washed with PBS. Left-atrial (LA) tissues were fixed in 4% paraformaldehyde, embedded in paraffin, sectioned in short-axis slices (4 μm thick), and stained on positively charged glass slides (Histobond+, Marienfeld). LA tissues from healthy (naïve) adult male SD rats were used as a reference for the baseline state in all stains.

IHC staining was performed using the BONDIII fully automated IHC staining system (Leica Biosystems, Buffalo Grove, IL, USA). IHC antigen retrieval was performed using BOND Epitope Retrieval Solution 2 (Tris-EDTA, prediluted, pH 8.0) for 20 min at 100°C. For LA fibrosis, the sections were stained with Masson's trichrome. For the detection and localization of SK4 channels in the working myocardium and endocardial tissue of rat hearts, we incubated the specimens with the primary antibody (IK1 D-5: sc-365265, Santa Cruz, mouse monoclonal antibody, 1:50) for 40 min, probed these samples with the secondary antibody for 30 min, and stained with 3,3′-diaminobenzidine (DAB) for 15 min (Bond Polymer refine detection kit, LE-DS9800, Leica biosystems). For the detection of α-SMA and NLRP3, we incubated the specimens with the primary antibodies specific for α-SMA (EPR5368, ab124964, abcam, rabbit monoclonal antibody against the N-terminus of human α-SMA, 1:1,000) and NLRP3 (bs-10021R, Bioss Antibodies, rabbit polyclonal antibody against human NLRP3, 1:200), respectively, for 40 min, probed them with an appropriate secondary antibody for 30 min, and labeled then with RED-alkaline phosphatase for 15 min (Bond Polymer Refine Red detection kit, LE-DS9390, Leica Biosystems). The specimens were then counterstained with hematoxylin and cover-slipped. The stained sections were left to dry for 24 h and scanned the following day using an Aperio slide scanner (Aperio Versa 200 DM6000 B, Leica Biosystems), after which they were analyzed using Qupath, a quantitative pathology and bioimage analysis software as described (23).

### Immunofluorescence

Antigen retrieval was performed using BOND Epitope Retrieval Solution 1 (Citrate buffer, prediluted, pH 6.0) for 20 min at 100°C, followed by peroxide treatment for 10 min. For membranal staining, we primarily incubated the sections with WGA (29022, CF488 WGA, Biotium, 1:500 in PBS) conjugated with Alexa Fluor 488 for 30 min, followed by incubation with blocking buffer (10% normal goat serum, 0.1% Triton, and 10% bovine serum albumin) for 30 min. For analyses of SK4 expression and localization, we incubated the specimens with the primary antibody (ALM-051, Alomone Labs, mouse monoclonal antibody against the 3rd extracellular loop of human SK4, 1:50) for 90 min and then stained with a Cy3-conjugated secondary antibody (711-165-151, Jackson Immunoresearch Laboratories, Cy^TM^3-conjugated AffiniPure Donkey Anti-Mouse, 1:100) for 90 min. For Cx43 expression and localization analyses, we incubated the specimens with the primary antibody (C6219, Sigma-Aldrich, rabbit polyclonal antibody against the C-terminus of human/rat Cx43, 1:400) for 90 min and stained with a Cy5-conjugated secondary antibody (711-175-152, Jackson Immunoresearch Laboratories, Cy^TM^5-conjugated AffiniPure Donkey Anti-Rabbit, 1:200) for 90 min. At the end of the staining, we incubated the specimens with Vector TrueVIEW Autofluorescence Quenching kit (SP-8400-15, Vector laboratories) for 10 min to improve the signal-to-noise ratio and reduce autofluorescence. Lastly, the specimens were stained with DAPI (D9564, Sigma-Aldrich, 1:1,000). The specimens were then cover-slipped with VECTASHIELD Vibrance Antifade Mounting (SP-8400-15, Vector laboratories), were left to dry for 24 h, and were scanned the following day. Prepared slides were visualized with a laser confocal microscope (Leica SP8. Leica Biosystems, Buffalo Grove, IL, 60089, USA) and the images were analyzed and processed using Qupath, ImageJ (National Institutes of Health and the University of Wisconsin, USA), and Adobe Illustrator (Adobe Systems, Inc., Mountain View, CA, USA).

### Histological analyses

Cardiac fibrosis and collagen deposition were measured by Masson's trichrome staining. LA myocardial and epicardial fibrosis were analyzed in the myocardium and epicardium of the entire section for a total of 18 images per animal. A predetermined threshold was selected, differentiating between normal and fibrotic tissue. Total fibrosis was calculated as the percentage of interstitial fibrosis in Qupath. SK4 channel, α-SMA, and NLRP3 expression were measured by IHC by implementing the same analysis methods. ECM accumulation was measured by WGA staining in left-atrial myocardial tissue 0.25 µm snapshots. The entire image was selected, with a total of 18 images per animal. A predetermined threshold was selected, measuring the amount of ECM in healthy and post-MI atrial tissue. For Cx43 lateralization analyses in atrial sections, only longitudinal sections were used. We performed immunofluorescent double staining using WGA and anti-Cx43. Lateralization was determined by comparing Cx43 localization to WGA staining of the membrane of longitudinal cardiomyocytes only. Lateralization results were presented and quantified as the ratio between Cx43 length (µm) when parallel/colocalized with the cell membrane (+SEM) to the cell's width + SEM (i.e. Cx43 length in the gap junction).

### Statistical analysis

A total of four naïve, seven vehicle-treated, and eight BA6b9-treated rats were used in the final analyses. Data analysis was performed using Prism 9.0 (GraphPad Software, Inc., San Diego, CA, USA). All data were expressed as the mean ± SEM (standard error of the mean). For these analyses, normality assumptions were tested using the Shapiro–Wilk test. Accordingly, unpaired Student's *t*-tests and one-way ANOVAs with Tukey's post hoc test were used for normally distributed results, whereas Mann–Whitney and Kruskal–Wallis tests with Dunn's multiple comparisons post-test were used for non-normally distributed results. Analyses comparing results from the same instrumented rats at 1 week vs. 4 weeks were performed using paired Student's *t*-test or Wilcoxon rank sum tests for normally and non-normally distributed results, respectively. Details of the specific statistical tests used are indicated in each figure/table. The threshold for significance was set at *P* < 0.05. The *P*-values are displayed graphically as follows: *P < 0.05, **P < 0.01, and ***P < 0.001.

## Supplementary Material

pgae192_Supplementary_Data

## Data Availability

All data generated or analyzed during this study are included in this published article.
