# Supplementary material for: The SK4 channel allosteric blocker, BA6b9, reduces atrial fibrillation substrate in rats with reduced ejection fraction
Source: PNAS Nexus. 2024 May 12;3(5):pgae192. doi: 10.1093/pnasnexus/pgae192 (PMC11114471; doi:10.1093/pnasnexus/pgae192)
Supplement: pgae192_Supplementary_Data [file pgae192_supplementary_data.docx]

**Supplemental data**

**The SK4 channel allosteric blocker, BA6b9, reduces atrial fibrillation substrate in rats with reduced ejection fraction**

Shira Burg^1^**^*^**, Or Levi^2,3^**^*^**, Sigal Elyagon ^2,3^, Shir Shapiro ^2,3^, Michael Murninkas ^2,3^, Sharon Etzion ^3^, Gideon Grawohl^4^, Daria Makarovsky ^5^ , Alexandra Lichtenstein ^5^, Bernard Attali ^1^, Yoram Etzion^2, 3^

**Affiliations:**

^1^ Department of Physiology & Pharmacology, Tel Aviv University, Tel Aviv 69978, Israel.

^2^ Cardiac Arrhythmia Research Laboratory, Department of Physiology and Cell Biology, Faculty of Health Sciences, Ben-Gurion University of the Negev, Beer-Sheva, Israel.

^3^ Regenerative Medicine & Stem Cell Research Center, Ben-Gurion University of the Negev, Beer-Sheva, Israel.

^4^ Medical Engineering Unit. The Jerusalem College of Technology, Jerusalem, Israel.

^5^ Inter Departmental Core Facility, Sackler Faculty of Medicine, Tel Aviv University, Israel

**
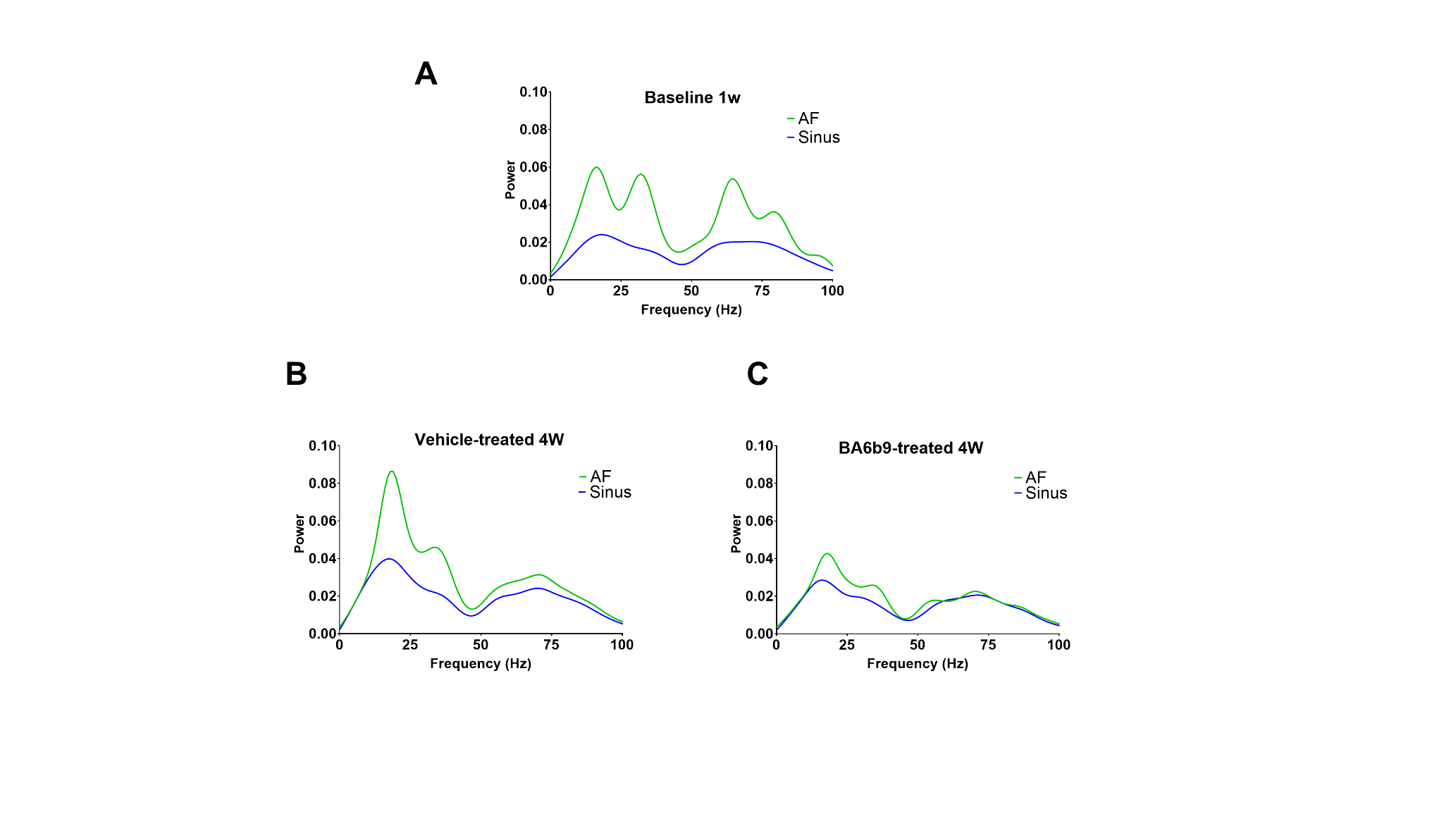
**

**Figure S1: Power spectrum analysis of atrial waveforms. A.** Mean power spectrum of the AF episodes (green) and pre-burst sinus rhythm (blue), at baseline (before randomization to a treatment arm). **B-C.** Similar results to those in (A) from the final EP study in vehicle-treated rats and BA6b9-treated rats, respectively.

.


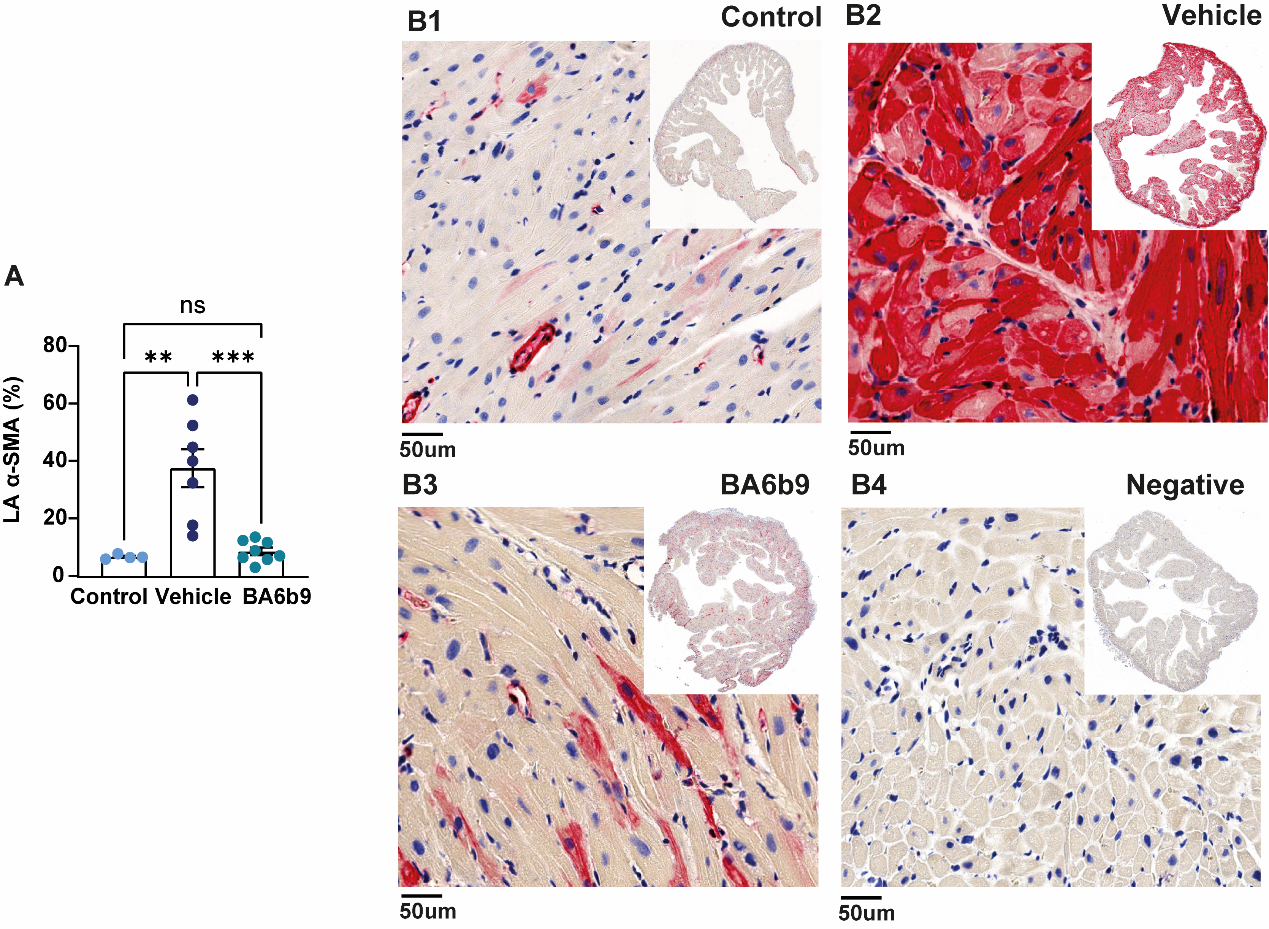


**Figure S2: Effect of BA6b9 on α-SMA expression in the left atrium of rats with MI-induced heart failure.** **A**. Statistical summary of entire left-atrial α-SMA expression; Vehicle vs. BA6b9 treated rats, compared to control (n=7, n=8, n=4, respectively; one-way ANOVA, F (2, 16) = 16.22, Sidak's multiple comparisons test P=0.0001). **B1.** Representative histological cross-section of a control rat LA (upper left), stained with Sirius Red. **B2-B3**. Representative Sirius Red-stained histological cross-sections of the LA in post-MI rats treated with vehicle (upper right) or BA6b9 (lower left) for 21 days. Positive red staining indicates α-SMA expression. **B4.** Negative control for α-SMA staining.


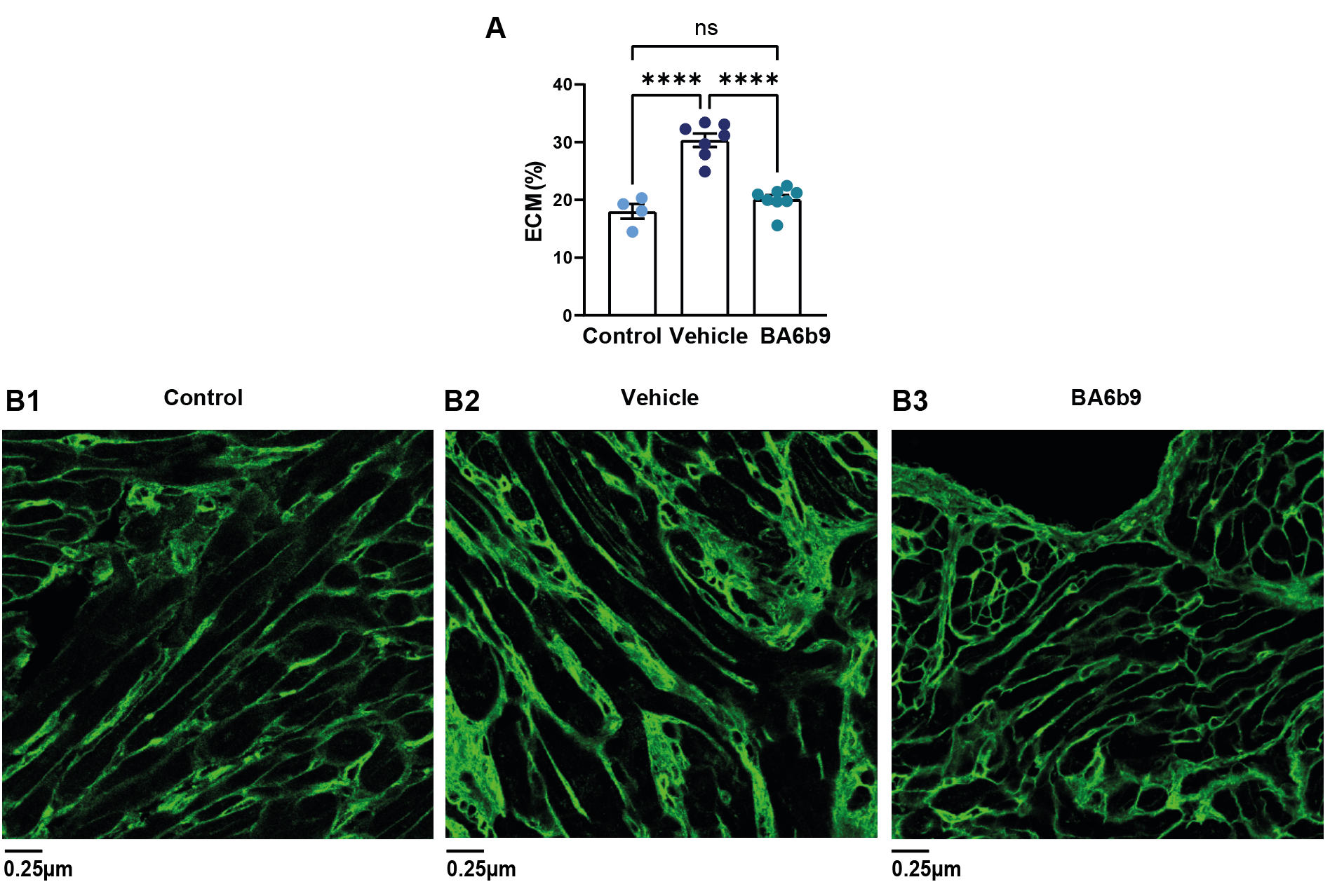


**Figure S3: Effect of BA6b9 on ECM accumulation in the atrial myocardium of rats with MI-induced heart failure. A**. Statistical summary of ECM accumulation in the left atrial myocardium (analysis of 6 randomized areas in the atrial myocardium for each atrial section, 18 total atrial-myocardial fields per animal). ECM accumulation was analyzed in immunofluorescent confocal images stained with wheat germ agglutinin (green) for membranal identification. The histogram compares control, vehicle-treated, and BA6b9-treated rats (n=4, n=7, n=8, respectively; one-way ANOVA, F (2, 16) = 40.48, Sidak's multiple comparisons test P<0.0001). **B1.** Representative section of the LA from a control rat (left), demonstrating thin membranal staining. **B2**. Representative section of the LA from a post-MI rat treated with vehicle (middle). Note the excessive ECM accumulation, leading to a marked thickening of the cardiomyocyte membrane compared to control rats. **B3**. Representative section of the LA from a post-MI rat treated with BA6b9 (right) for 21 days. Note the decrease in membranal thickening, more closely resembling the control rat. ECM: extracellular matrix.

**
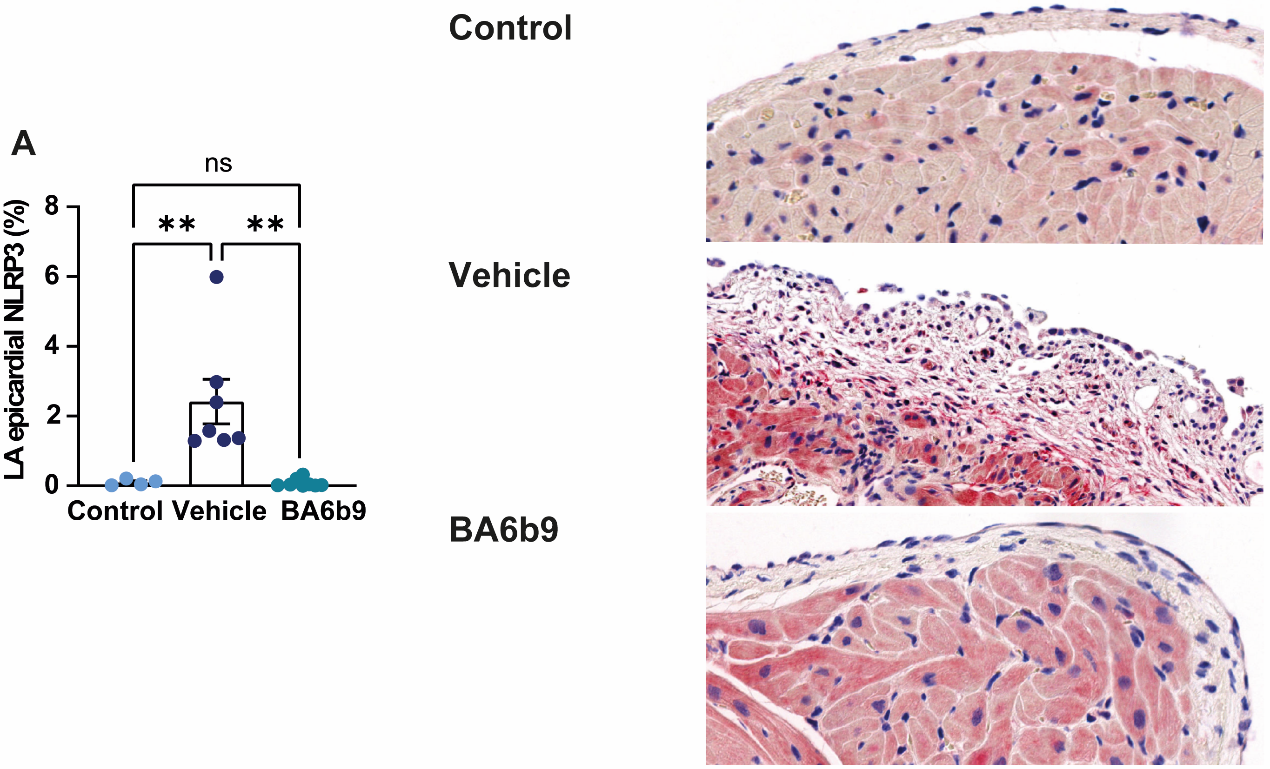
**

**Figure S4: Effect of BA6b9 on NLRP3 expression in the left atrial epicardium of rats with MI-induced heart failure. A.** Statistical summary of LA epicardial NLRP3 expression; (analysis of 6 randomized epicardial fields for each atrial section, 18 total atrial-epicardium fields per animal); control, vehicle-treated, and BA6b9-treated rats were compared (n=7, n=8, n=4, respectively; one-way ANOVA, F (2, 16) = 10.97, Sidak's multiple comparisons test P=0.0010). **Right panel:** Representative Sirius Red-stained histological cross-sections of the LA myocardium attached to the epicardial tissue from a control rat (upper), a post-MI rat treated with vehicle (middle), and a rat treated with BA6b9 (lower).
